# A computational insight on the aromatic amino acids conjugation with [Cp*Rh(H_2_O)_3_]^2+^ by using the meta-dynamics/FMO3 approach

**DOI:** 10.1007/s00894-023-05794-z

**Published:** 2023-12-12

**Authors:** Roberto Paciotti, Alessandro Marrone

**Affiliations:** Department of Pharmacy, Università “G. D’Annunzio” Di Chieti-Pescara, I-66100 Chieti, Italy

**Keywords:** FMO3, Pair interaction energy, MTD, Meta-dynamics, Metal-based drugs, [Cp*Rh]^2+^

## Abstract

**Context:**

Rh(III) complexes demonstrated to exert promising pharmacological effects with potential applications as anti-cancer, anti-bacterial, and antimicrobial agents. One important Rh(III)-ligand is the pentamethylcyclopentadienyl (Cp*) group forming in water the [Cp*Rh(H_2_O)_3_]^2+^ complex. Among of its attractive chemical properties is the ability to react specifically with Tyr amino acid side chain of G-protein–coupled receptor (GPCR) peptides by means of highly chemoselective bioconjugation reaction, at room temperature and at pH 5–6. In this computational work, in order to deepen the mechanism of this chemoselective conjugation, we study the ligand exchange reaction between [Cp*Rh(H_2_O)_3_]^2+^ and three small molecules, namely p-cresol, 3-methylimidazole, and toluene, selected as mimetic of aromatic side chains of tyrosine (Tyr), tryptophan (Trp) and phenylalanine (Phe), respectively. Our outcomes suggest that the high selectivity for Tyr side chain might be related to OH group able to affect both thermodynamic and kinetic of ligand exchange reaction, due to its ability to act as both H bond acceptor and donor. These mechanistic aspects can be used to design new metal drugs containing the [Cp*Rh]^2+^ scaffold targeting specifically Tyr residues involved in biological/pathological processes such as phosphorylation by means of Tyr-kinase enzyme and protein–protein interactions.

**Methods:**

The geometry of three encounter complexes and product adducts were optimized at the B3LYP//CPCM/ωB97X-D level of theory, adopting the 6-311+G(d,p) basis set for all non-metal atoms and the LANL2DZ pseudopotential for the Rh atom. Meta-dynamics RMSD (MTD(RMSD)) calculations at GFN2-xTB level of theory were performed in NVT conditions at 298.15 K to investigate the bioconjugation reactions (simulation time: 100 ps; integration step 2.0; implicit solvent model: GBSA). The MTD(RMSD) simulation was performed in two replicates for each encounter complex. Final representative subsets of 100 structures for each run were gained with a sampling rate of 1 ps and analyzed by performing single point calculations using the FMO3 method at RI-MP2/6-311G//PCM[1] level of theory, adopting the MCP-TZP core potential for Rh atom.

**Supplementary Information:**

The online version contains supplementary material available at 10.1007/s00894-023-05794-z.

## Introduction

The vast chemical complexity of biological systems is probably the most challenging aspect to deal with in the development of new therapeutics. Indeed, the capability of exerting an atomistic control of the structure and function of biomolecular systems via the administration of active compounds is often hampered by the lack of an adequate chemical and/or compartmental/tissue selectivity.

In this frame, the high toxicity disclosed by metal-based drugs can be often ascribed to their high and multifacet reactivity inducing an uncontrolled multi-target or multi-organ response instead of specific therapeutic activity.

Since the discovery of cisplatin, CP [1], the search for new effective metal drugs is a never-ending race. The anticancer activity of this prominent metallodrug is ascribed to the binding at consecutive purine nucleobases by causing so-called 1,2-intrastrand lesions. Nevertheless, together with its derivatives (carboplatin and oxaliplatin), CP lasts as the most used metal drug for the treatment of several solid tumors [2, 3], in spite of its nephrotoxicity and chemoresistance [4, 5].

As a consequence, several efforts were performed to discover new classes of metal-drugs containing other metals such as Au, Ti, Ru, Re, Ga, Ir, Rh, and others [6–8].

Among them, Rh(III) complexes, compared with the typical square planar Pt(II) complexes, is characterized by structural diversity which is considered as a potential advantage of this metal ion which leads to the different modes of interaction of these complexes with biomolecular targets [9]. They have shown several pharmaceutical applications such as anti-cancer, anti-bacterial, and antimicrobial activity acting as protein binders or modulating protein–protein interactions (PPIs) [9]. Notably, Rh-containing complexes can be used not only for therapeutic applications but also as luminescent probes or labels for biomolecules [10].

Due to these important pharmacological properties, Rh-containing compounds were deeply studied and many complexes were designed with promising in vitro efficacy [11, 12] although none of them have reached the market.

One important Rh(III)-ligand is the pentamethylcyclopentadienyl (Cp*) group since it gives favorable chemical properties to the corresponding metal complexes such as a good solubility and stability [13]. Due to these important features, [Cp*Rh]^2+^ complexes have widely applied in catalysis [14, 15] and in coordination chemistry.

Many Rh(III) complexes containing Cp* were designed to act as isomerase inhibitors demonstrated promising in vitro activity [9]. [Cp*Rh]^2+^ scaffold is also included in metal compound, such as [Cp*Rh(Ph_3_P)Cl_2_], which act as protease inhibitors with potential therapeutic use for the treatment of diseases such as cancers, fungal and viral infections, Alzheimer’s disease, and inflammatory disorders [9]. Interestingly, the [Cp*Rh]^2+^ complexes were also investigated as possible vehicles to deliver anticancer drugs, such as curcumin, to the tumor cells [16].

In water the [Cp*Rh]^2+^ complexes is found in the hydrated form, [Cp*Rh(H_2_O)_3_]^2+^. It is able to react specifically with Tyr amino acid side chain of G-protein–coupled receptor (GPCR) peptides, as [Tyr^1^]-Leu-enkephalin, [Tyr^4^]-neurotensin (8-13), and [Tyr^3^]-octreotide, by means of highly chemoselective bioconjugation reaction, at room temperature and at pH 5-6 [17, 18]. In a recent computational study, performed using classical molecular dynamics (MD) and density functional theory (DFT) methods, we analyzed the effect of Tyr conjugation reactions on protein conformations of the above-mentioned GPCR peptides [19]. Moreover, we found that the mesomeric e-donating (+M) effect of the Tyr phenol OH group provides high thermodynamic stability to [Cp*Rh(Tyr-peptide)]^2+^ complex, representing the mechanistic rationale for this chemoselective reaction.

**Fig. 1:**
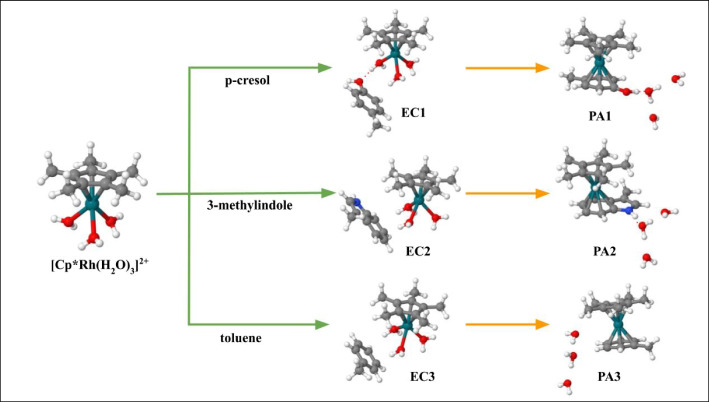
Schematic representation of ligand exchange reaction involving [Cp*Rh(H_2_O)_3_]^2+^ and three small molecules selected as mimetic of aromatic a.a. side chain as p-cresol (**pC**), 3-methylindole (**3MI**), and toluene (**T**). The conjugation reactions, that starting from encounter complexes (ECs) lead to product adducts (PAs), are reported by using orange arrows

In this work, in order to deepen the mechanism of this chemoselective conjugation we study the ligand exchange reaction between [Cp*Rh(H_2_O)_3_]^2+^ and three small molecules, namely p-cresol (**pC**), 3-methylimidazole (**3MI**), and toluene (**T**), selected as mimetic of aromatic side chains of Tyr, Trp and Phe, respectively, by using meta-dynamics (MTD) [20] and the ab initio fragment molecular orbital (FMO) method [21]. As shown in Fig. 1, starting from the encounter complexes (EC), where the a.a. mimetic (X) interacts with [Cp*Rh(H_2_O)_3_]^2+^ by non-covalent interactions, the ligand exchange reactions was promoted by adopting the MTD protocol obtaining the corresponding product adduct (PA), {[Cp*Rh(η^6^-X)]^2+^ ・3H_2_O}, where the three water molecules surround the η^6^-complex.

The conjugation reactions were analyzed by using the FMO method which allows splitting the system in fragments and then computing the total energy as function of the pair interaction energies (PIEs) [22]. Moreover, the energy decomposition analysis (EDA) [23, 24] of each PIE provides important information on the nature of the interactions between fragments, such as X and [Cp*Rh]^2+^ scaffold. Thus, MTD/FMO method allowed to characterize and monitor the evolution of conjugation reaction giving a new insight on this high chemoselective process and results has been used to suggest potential applications of Rh(III) metal drugs containing the [Cp*Rh]^2+^ scaffold.

## Methods

The structures of **EC1**, **EC2,** and **EC3**, retrieved from the previous study [19], were optimized at the B3LYP level of theory by using the all-electron 6-311+G(d,p) basis set for all non-metal atoms. The Rh atom was treated with the LANL2DZ basis set which includes the LANL2 pseudopotential to describe the core electrons and a Hay and Wadt double-zeta basis set to describe the valence shell electrons. Vibrational frequency calculations were then performed at the same level of theory to estimate the zero point energy (ZPE), the thermal corrections, and the gas phase vibrational entropies. The same scheme was employed to optimize the structures of the corresponding *η*^6^-complexes. The effect of explicit water on the stability of these complexes was also evaluated by including one, two and three water molecules obtaining the corresponding hydrated complexes which were optimized at same level of theory. Then, the single point energy of all the optimized geometries was calculated with ωB97X-D functional (same basis set employed in the geometry optimization) and simulating the water effect with CPCM method. Indeed, the ωB97X-D hybrid functional has been demonstrated to reproduce with good accuracy the energy of several metal complexes [25–27], and therefore, it was here employed to assess the relative stability of conjugated complexes. The free energies (G) and enthalpies (H) computed at B3LYP//CPCM/ωB97X-D level of theory were obtained as follows:

1 $$G={E}_{\textrm{sol}}+{G}_{\textrm{therm}}$$

2 $$H={E}_{\textrm{sol}}+{H}_{\textrm{therm}}$$

where *E*_sol_ is the electronic energy calculated at CPCM/ωB97X-D level of theory, and *H*_therm_ and *G*_therm_ are the sum of several correction terms: zero-point energy, enthalpy, and entropy corrections (included only in *G*_therm_), computed at B3LYP level of theory in gas phase.

All DFT calculations were performed with Gaussian 09 package [28].

Meta-dynamics RMSD (MTD(RMSD)) [29] calculations at GFN2-xTB level of theory [30], implemented in xTB software [31], were performed to model the EC⟶PA process (vide infra). The standard root-mean-square deviation (RMSD) in Cartesian space was used to define the collective variables (CVs). Here, the oxygen atoms of the three water molecules were considered to initialize the RMSD criteria, and define the CVs that pushed these three water molecules away from the Rh atom during the simulations — and allowed the coordination of the aromatic fragment on the metal center. The scaling factor for RMSD criteria (*k*_push_) was set to 0.05 with a width of the Gaussian potential (alpha) used in the RMSD criteria of 0.9.

All systems were simulated for 100 ps using the GBSA implicit solvent model, in NVT conditions at 298.15 K, with an integration step 2.0 fs. Each MTD trajectory was composed by sampling one snapshot per 50.0 fs, and a final representative subset of 100 structures for each run was finally gained with a sampling rate of 1 ps. The MTD(RMSD) simulation was performed in two replicates (two runs) for each EC.

In order to assess the stability of each EC, we also performed 50 ps MD calculations for each of them, by essentially adopting the same set-up scheme but without applying the MTD(RMSD) protocol.

To avoid that non covalent binding molecules move away during MTD and MD simulations, all the EC structures were confined by a repulsive spherical potential (potential = logfermi; sphere: auto, all).

The representative structures of MTD(RMSD) and MD trajectories were then minimized at GFN2-xTB level of theory, using the GBSA model to simulate the solvation effect.

The optimized geometries resulting from either MTD or MD studies were then analyzed by using the fragment molecular orbitals (FMO) methodology and adopting the three-body approach (FMO3) [32]. Notably, although the FMO method is widely applied for the investigation of interactions involving large biological systems as proteins structure [33, 34], protein–protein interaction [35, 36], protein–DNA interaction [37] and ligand-receptor complexes [38, 39], it can be used also to study small metal complexes as previously done [40]. Here, the FMO method can be profitably used to monitor the ligand exchange reactions in terms of pair interaction energies (PIEs) because the composition of the fragments is not altered by the breaking/formation of coordinate bonds during reaction.

All FMO calculations were performed at RI-MP2/6-311G level of theory [41] adopting the triple-ζ model core potential (MCP-TZP) for the Rh atom [42]. The water solvation effect was simulated through the PCM[1] method, by computing the repulsion and dispersion contributions by the empirical method of Floris and Tomasi [43], using a high density of tesserae on the cavity surface (NTSALL = 240) and FIXPVA as tessellation scheme [44]. The solvent screening effect was simulated using the partial screening method (MODPAR=73) [45]. To prevent overestimation of charge transfer energy [46, 47], the screened point charges were adopted and the ESP-PTC was computed using the charge damping [48] for all atoms with a=b=1 (SCREEN=1,1; RESPPC = −1).

**Fig. 2:**
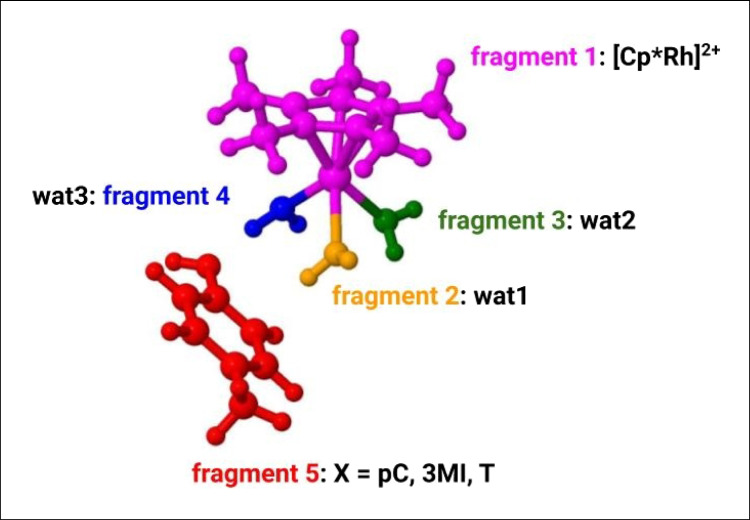
Fragmentation scheme employed in FMO calculations where X is **pC**, **3MI**, and **T**. [Cp*Rh]^2+^, H_2_O (wat1), H_2_O (wat2), H_2_O (wat3), and X were included in fragments 1 (magenta), 2 (orange), 3 (green), 4 (blue), and 5 (red), respectively

The structures were split in five fragments as shown in Fig. 2: [CP*Rh]^2+^ (fragment 1), three distinct fragments each one including one water molecule (fragments 2, 3, and 4) and X (fragment 5).

The FMO calculations also included the energy decomposition analyses (EDA) with FMO3 corrections [49] in which each PIE value is decomposed as follows:

3 $${\textrm{PIE}}_{\textrm{IJ}}={E}^{\textrm{es}}+{E}^{\textrm{ex}}+{E}^{\textrm{ct}}+{E}^{\textrm{disp}}+{E}_{\textrm{solv}}$$

where the terms *E*^es^, *E*^ex^, *E*^ct^, *E*^disp^, and *E*_solv_ refer to electrostatic, exchange repulsion, charge transfer, dispersion and solvation energies, respectively. These terms provide important information on the nature of the interfragment interactions and disclose the energy components playing the main role in the binding process. The *E*^es^ component is related to Coulomb interaction between charged or polarized fragments. The *E*^ex^ term is always repulsive and can be ascribed to steric repulsion between close fragments. The *E*^ct^ component is related to the interaction between occupied orbitals of a donor and unoccupied orbitals of an acceptor. The dispersion energy term, *E*^disp^, takes into account the contribution of the interaction between the temporary dipole moments of two fragments, especially important for residues not exposed to the solvent and involved in the hydrophobic interactions [50].

In analogy with ligand-receptor study [46], an estimation of the stability of the ECs is provided by the sum of PIEs between X and all fragments of [Cp*Rh(H_2_O)_3_]^2+^ (fragments 1, 2, 3, and 4):

4 $${E}^{\textrm{INT}}=\sum_{i=1}^4{PIE}_{Xi}$$

For each EC and PA structure, we also computed the total PIE (TPIE), i.e., the sum of all PIEs within the structure:

5 $$\textrm{TPIE}={\sum}_{i>j}^N PI{E}_{ij}$$

All FMO calculations were performed by using the GAMESS-US package (version: 30 June 2021 — R1) [51].

## Results

### Thermodynamics of PAs formation

The thermodynamics of EC⟶PA processes was preliminary assessed at DFT level of theory (see above). All the possible configurations of the three water molecules in each of the **PA1-3** complexes were manually built and minimized. The most stable structures were then used to calculate the free energy and enthalpy values for the EC⟶PA reaction (Fig. 1 and S1).

All examined processes were characterized by negative ΔG (Table 1) suggesting that the ligand exchange reaction, EC ⟶ PA, is thermodynamically favorable for each of the X ligands. The most negative value was computed for the **PA1** formation with the following order ΔG1 < ΔG2 < ΔG3 indicating that the **pC** is able to replace the three water molecules with more efficiency than **3MI** and **T**. This result is in good agreement with our previous study where we estimated the same trend, **pC** > **3MI** > **T**, for the formation of η^6^-[Cp*Rh(X)]^2+^ complex [19].

**Table 1:** ΔG, ΔH, and relative ΔH (ΔH_rel_) of the three ligand exchange reaction, **EC1** ⟶ **PA1**, **EC2** ⟶ **PA2**, **EC3** ⟶ **PA3** investigated in this work, computed at B3LYP/6-311+G(d,p)//ωB97X-D/6-311+G(d,p). The total pair interaction energies (ΔTPIE) are reported along with the corresponding relative values. All energy values are in kcal/mol

Reaction	ΔG	ΔH	ΔH_rel_	ΔTPIE	ΔTPIE_rel_
*EC1 ⟶ PA1*	−14.9	−14.6	0.0	−25.1	0.0
*EC2 ⟶ PA2*	−12.7	−9.7	4.9	−19.5	5.6
*EC3 ⟶ PA3*	−7.6	−7.8	6.8	−17.4	7.6

With exception of reaction **EC2** ⟶ **PA2**, the reaction ΔG values are appreciably close to the corresponding ΔH values, presumably because of the small vibrational entropy variation accompanying this substitution process. Thus, the exergodicity of the EC⟶PA process estimated by our approach is mostly determined by the reaction enthalpy that quantifies the energy gain for the replacement of O⟶Rh with π⟶Rh bonds and for the formation of hydrogen bonds between the released water molecules.

In the framework of application of the FMO method, the E^INT^ has been used as an estimation of reaction ΔH, for example in receptor-ligand binding processes [52]. The ΔH of a reaction, Δ_r_H, can be also computed as difference between the sum of enthalpy variation of formed bonds and the sum of ΔH of the broken bonds. Thus, considering that in EC ⟶ PA reaction there are only ligands exchange without breaking of pure covalent bonds, the ΔH of such reaction can be estimated in principle by considering the difference between PIE values of the broken and formed ligands interactions passing from EC to PA (ΔTPIE):

6 $${\varDelta}_{\textrm{r}}\textrm{H}=\left[\sum \varDelta \textrm{H}\left(\textrm{formed}\ \textrm{bonds}\right)-\sum \varDelta \textrm{H}\left(\textrm{broken}\ \textrm{bonds}\right)\right]\approx \varDelta \textrm{TPIE}={\textrm{TPIE}}_{\textrm{PA}}-{\textrm{TPIE}}_{\textrm{EC}}$$

where TPIE_PA_ and TPIE_EC_ are the total pair interaction energies computed according Eq. (5) for PA and EC, respectively.

As reported in Table 1, ΔTPIE terms reproduce quite well the trend of ΔH values (they are all 10 kcal/mol higher in energy), especially the relative values with an error < 1 kcal/mol, suggesting that the FMO method might be used to estimate the ΔH of EC⟶PA reaction and compare the ligand exchange efficiencies of **pC**, **3MI,** and **T**.

### MTD/FMO descriptions of PAs formation

To explore the EC⟶PA reaction path, we simulated each encounter complex (two production runs of 100 ps per EC) using the MTD(RMSD) protocol implemented in xTB. The pushing potential was applied only to the oxygen atoms of the coordinated water molecules. Being the system simulated into a spherical confinement and implicit solvation, the gradually decomplexated water molecules last in proximity of the system, thus, potentially influencing the approach and coordination of the X ligand at the metal fragment. To assay the impact of non-coordinated water molecules on the stability of the system, the geometry of the [Cp*Rh(X)]^2+^, {[Cp*Rh(X)]^2+^・H_2_O}, {[Cp*Rh(X)]^2+^・(H_2_O)_2_}, and {[Cp*Rh(X)]^2+^・(H_2_O)_3_} were optimized at DFT level of theory by testing several configurations of explicit water molecules. Then, the most stable structures (Fig. S1) were analyzed with the FMO3 scheme [1].

**Fig. 3:**
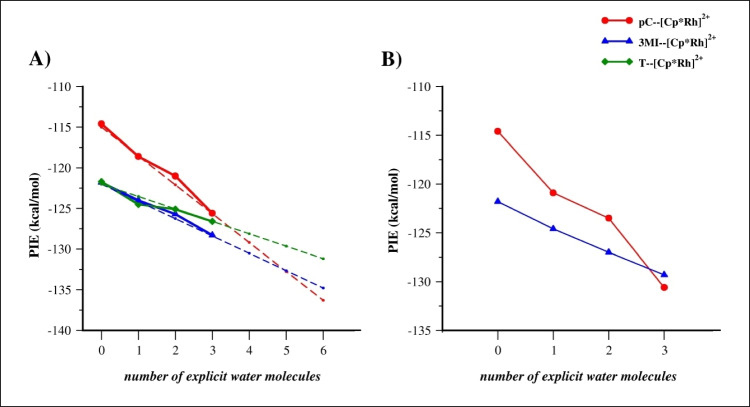
PIE values of pair formed by X and [Cp*Rh]^2+^ scaffold by varying the number of explicit waters (from zero to three) **A)** including X, [Cp*Rh]^2+^, and water molecules in separated fragments and **B)** including X and water directly interacting via H bond in the same fragment. In Fig. 3A, the trend lines are also reported

As shown in Fig. 3A and Table S1, the X--[Cp*Rh]^2+^ PIEs without including explicit water molecules are almost the same for **3MI** and **T** (−121.8 and −121.7 kcal/mol, respectively) while a less negative value was found for **pC** (−114.6 kcal/mol). Including one, two, and three explicit water molecules the X--[Cp*Rh]^2+^ PIE decreases (more negative values). The curves related to pC--[Cp*Rh]^2+^ and 3MI--[Cp*Rh]^2+^ PIEs significantly decrease indicating that the interactions with explicit waters affect mainly the two scaffolds able to establish H bonds with water which increase the electron density on the aromatic rings. Notably, the pC--[Cp*Rh]^2+^ PIE is characterized by the steepest curve although the X--[Cp*Rh]^2+^ PIE still follows the trend **3MI** > **T** > **pC**. Interestingly, by assuming an almost linear trend of PIE decrease and the cooperative effect of H bonds, we estimated that **T** > **3MI** > **pC** trend of X--[Cp*Rh]^2+^ PIE is gained with six explicit water molecules (Fig. 3A). Because the accuracy of FMO calculations increases by increasing the size (number of atoms) of fragments, a second set of FMO calculations were performed on the systems with X=**3MI** and **pC** by including the H-bond interacting water molecule to the latter fragments and evaluating the corresponding X--[Cp*Rh]^2+^ PIE values (Fig. 3B, Table S1).

Although the PIE of pC--[Cp*Rh]^2+^ was found to be larger compared to 3MI--[Cp*Rh]^2+^ in the system with no explicit water, the former decreases more steeply and becomes lower than the 3MI--[Cp*Rh]^2+^ PIE when three water molecules were considered (−130.6 and −129.3 kcal/mol, respectively). Thus, to correctly reproduce the interaction strength of the three aromatic mimetics of a.a. side chains by using the FMO3 method the inclusion of at least three explicit water molecules are needed and that one directly involved in H bond with aromatic scaffold should be included in its same fragment.

**Fig. 4:**
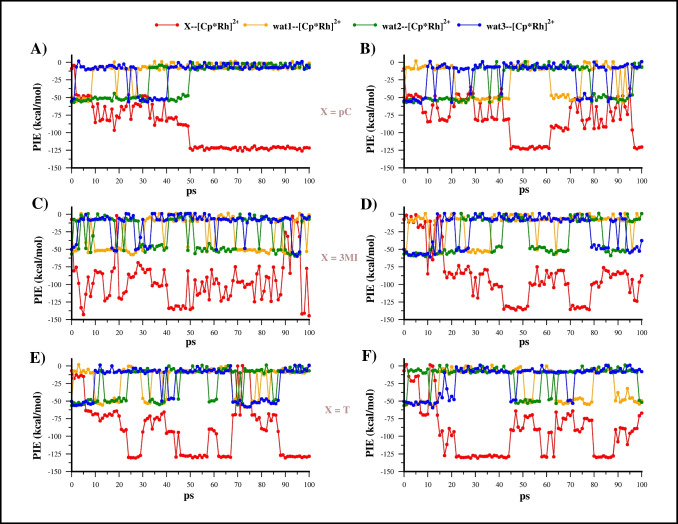
PIE values of fragments interacting with [Cp*Rh]^2+^ computed for the sampled structures of MTD(RMSD) run1 and run2 of **EC1** (**A** and **B**), **EC2** (**C** and **D**), and **EC3** (**E** and **F**)

The PIE values of all the fragments coordinated to the [Cp*Rh]^2+^ scaffold, namely X--[Cp*Rh]^2+^, wat1--[Cp*Rh]^2+^, wat2--[Cp*Rh]^2+^, wat3--[Cp*Rh]^2+^, for each X ligand, and retrieved from the corresponding MTD trajectories are reported in Fig. 4.

For X = **pC** (Fig. 4A and B), the aromatic ligand displaced the first water molecules (wat3, blue line) in a few ps. In the first MTD run (Fig. 4A), the second water molecules (wat1, orange profile) was replaced at 10 ps with consequent increasing of pC--[Cp*Rh]^2+^ interaction (more negative value of PIE). A similar trend of substitution of the first and second water was observed also in the second run (Fig. 4B). The last water molecule is released around 50 ps of the first run with formation of the η^6^-complex, which remains stable until the end of simulation (100 ps). Notably, in this segment of trajectory the PIE of X--[Cp*Rh]^2+^ assumes an average value of −122.0 ± 1.6 kcal/mol (Table S2) while the PIE between water molecules and [Cp*Rh]^2+^ are close to zero indicating that they are not bound to the metal scaffold. Whereas in second run (Fig. 4B), the η^6^-complex is formed before 50 ps although, at about 60 ps, one water molecule (wat1) came back to coordinate at [Cp*Rh]^2+^ breaking the η^6^ coordination of **pC** for 20 ps before its restoration at the end of trajectory.

The formation of [Cp*Rh(η^6^-X)]^2+^ complex was also detected before 50 ps in both runs of **3MI** and **T**, but these complexes lasted for a shorter time compared to **pC** (Fig. 4C–F). The average 3MI--[Cp*Rh]^2+^ and T--[Cp*Rh]^2+^ PIE in the η^6^ coordination were −130.4 ± 11.8 and is −128.8 ± 1.1 kcal/mol; hence, slightly more negative compared to pC--[Cp*Rh]^2+^ PIE (Table S2).

The energy decomposition analyses (EDA) of the X--[Cp*Rh]^2+^ PIE calculated in the MTD(RMSD) simulations were also performed. As shown in Fig. S2, *E*^es^ and *E*^ct^ are the terms that mostly contribute to determining negative PIE values. *E*^sol^ and *E*^ex^ are always positive terms and reach the highest values in η^6^ coordination due to the steric clashes between methyl groups of Cp* and aromatic scaffold.

We also computed the average values of the EDA terms for all examined η^6^-complexes in the MTD trajectories, and the results are reported on Table S2. Notably, the average *E*^ct^ values followed the **pC** < **3MI** < **T** trend, and the most negative value for **pC** (-130.9 kcal/mol) compared with **3MI** and **T** (−127.7 and −128.3 kcal/mol, respectively). Therefore, we observed that the trend of CT from X to [Cp*Rh]^2+^ over all the trajectory is complementary to the FMO3 charge of [Cp*Rh]^2+^ fragment, Q_3_, whose decrease from +2 to ~ +1.5 is mainly due to the CT from X (Fig. S3–S4).

**Fig. 5:**
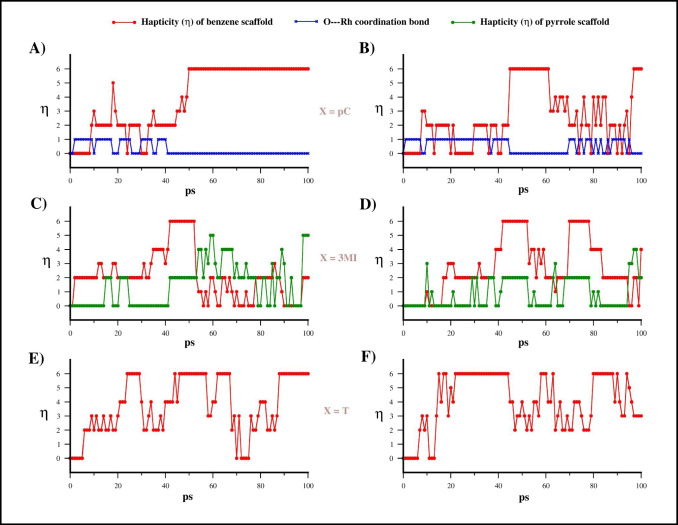
Estimation of hapticity of X--[Cp*Rh]^2+^ interaction computed on the base of C-Rh distance for structures of MTD(RMSD) run 1 and 2 for **EC1** (**A** and B), **EC2** (C and D), and **EC3** (E and F). For **EC1** (A and B) and **EC2** (C and D), the presence of a chemical bond between phenolic O and pyrrolic N atoms with Rh are reported by using blue and green lines, respectively. We assumed a C-Rh or N-Rh bond formed when the distance between C/N and Rh is below to 3 Å while the OH (**pC**) is considered bounded to Rh when O-Rh distance is below 2.5 Å

The hapticity (η) variation of the X scaffold during the reaction path was evaluated by computing the distance between Rh and C atoms of aromatic scaffold (**pC**, **3MI**, and **T**) along all the trajectories (Fig. 5). In the case of **pC** and **3MI,** the O-Rh and N-Rh distances, respectively, were also monitored.

**Fig. 6:**
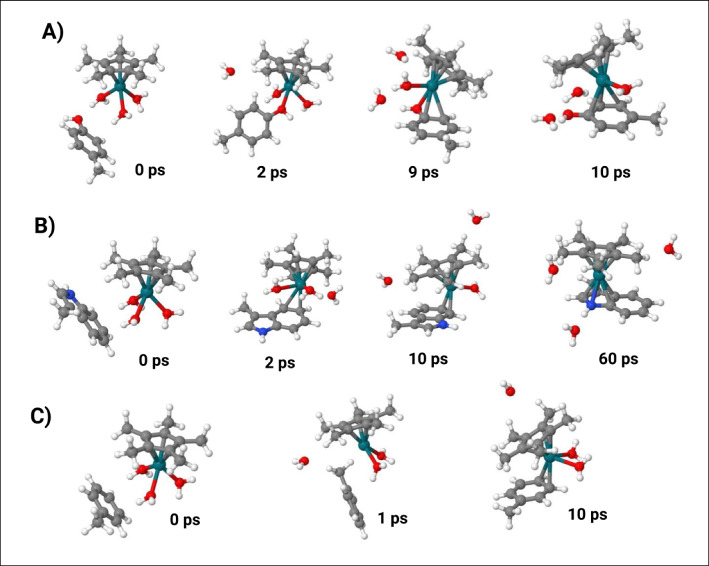
Most relevant structures of the first 10 ps of MTD(RMSD) run1 for **A)** **EC1**, **B) EC2**, and **C) EC3**

This analysis provides an interesting result for **pC** ligand. Indeed, the metal coordination of OH anticipates the replacement of the first water molecule by means of C atoms of benzene scaffold at the beginning of the trajectory of run1 (Figs. 5A and 6A). Then, around 9 ps, C_1_ and C_2_ of the benzene ring (Figs. 6A, 9 ps) start to coordinate the Rh ion leading to a peculiar η^3^-complex with the OC_1_C_2_ coordination of the phenol moiety. This configuration remains stable for ca 30 ps before the O-Rh interaction is replaced by other aromatic C atoms leading to the transient η^4^ intermediate that rapidly evolves in the η^6^-complex. This trend was also observed in run2 (Fig. 5B), where the simultaneous coordination of Rh by means of OH and two aromatic C atoms is observed also in the 70–95 ps segment of the trajectory. Thus, these results indicate that the OH group plays a crucial role, especially in the early stages of the interaction between **pC** and [Cp*Rh(H_2_O)_3_]^2+^ complex.

The analysis of C-Rh distances of reaction involving the **EC2** indicates that, in first run, the first leaving water molecule is replaced by atom C_4_ and C_5_ of the indole leading to the η^2^-complex (Figs. 5C and 6B). In about 37 ps, the hapticity of the phenyl ring becomes transiently η^4^ and then η^6^ in a few ps. Interestingly, at around 55 ps, the [Cp*Rh]^2+^ moiety starts interacting with the C_2_ and C_3_ of the pyrrolic portion of **3MI**, reaching the η^5^ at 60 ps (Fig. 6B). Then, the [Cp*Rh]^2+^ scaffold moves from pyrrolic to phenyl ring during in the later stages of the trajectory. Conversely, in the second run (Fig. 5D), we observed the involvement the phenyl ring only in the Rh-coordination of **3MI**. Notably, in both runs, the η^4^ coordination of **3MI** is maintained only for a few steps in the transit between η^2^ and η^6^ coordination.

The same scenario was found also for **T** complexes where the most recurring coordination motives are η^2^ and η^6^ while η^4^ is only transiently detected. The analysis of C-Rh distance reveals that, in spite of the early detachment of the first water molecule, the η^2^ coordination of **T** at Rh is not concomitant, as shown in Figs. 5E and F and 6C. Indeed, in the early 10 ps of the first run, **T** interacts with Rh only via weak electrostatic interaction of π-electrons of C_1_-C_2_ bond which rapidly evolves in η^2^ coordination by means of C_3_ and C_4_ (Fig. 6C). This evidence suggests a certain difficulty for **T** to initiate the interaction with Rh which should lead to the water displacement.

Notably, at 70 ps, **T** loses the contact with Rh restoring for few ps the [Cp*Rh(H_2_O_3_)]^2+^ complex (Fig. 5E), although this event was observed only in run1. Indeed, in the second run, **T** interacts with Rh for the entire trajectory with continuous interchanging between η^2^ and η^6^ coordination.

### MD/FMO analysis of ECs stability

In analogy with ligand-receptor studies [26], the interaction energy between X and [Cp*Rh(H_2_O)_3_]^2+^ can be estimated as the overall interfragment PIE named E^INT^ (Eq. 4). The computed *E*^INT^ values for **EC1**, **EC2**, and **EC3** are −17.5, −14.4, and −10.3 kcal/mol, respectively, suggesting that **pC** might effectively form a more stable EC with [Cp*Rh(H_2_O)_3_]^2+^ than **3MI** and **T** (Table S3).

**Fig. 7:**
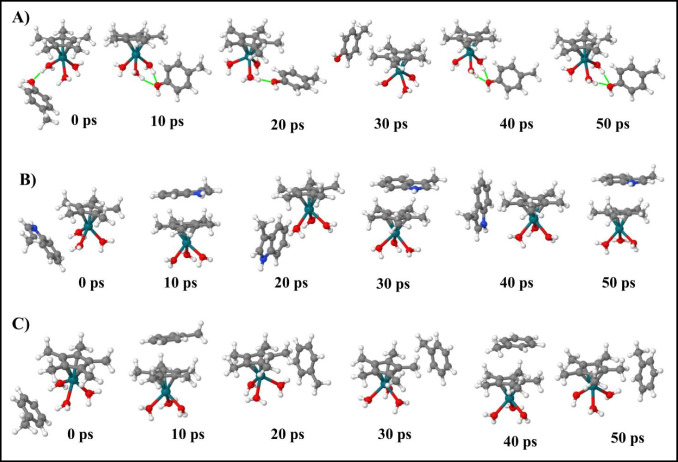
Representative structures of MD trajectories obtained without applying the MTD(RMSD) protocol starting from **A) EC1**, **B) EC2**, and **C) EC3** collected at 0, 10, 20, 30, 40, and 50 ps

**Fig. 8:**
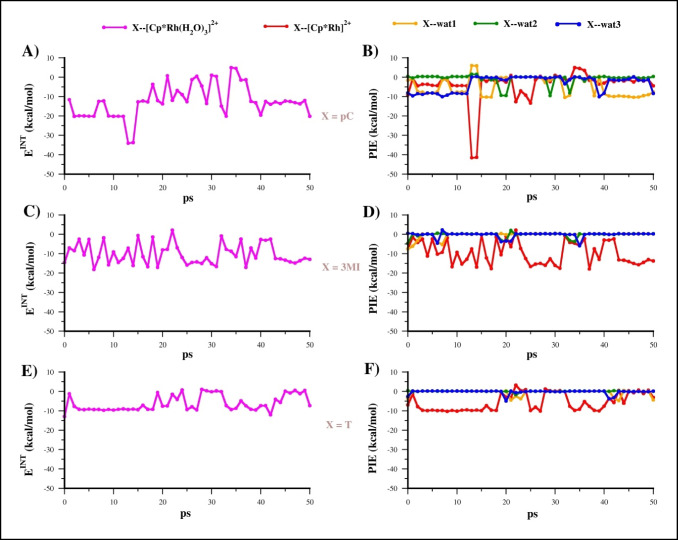
E^INT^ profiles computed for **A) pC**, **C) 3MI**, and **E) T** along with the corresponding PIE components (figures **B**, **D**, and **F**, respectively)

Thus, considering that the formation of a stable EC is an important checkpoint of the conjugation process, we performed 50 ps MD simulations, to follow up the formation of **EC1-3** complexes in native condition, i.e., without using the MTD(RMSD) protocol. The snapshots obtained by sampling each 10 ps of the **EC1**, **EC2**, and **EC3** trajectories are reported in Fig. 7, while the corresponding FMO analysis was summarized in Figs. 8 and S6.

The **pC** ligand establishes H bond interactions with one or two water molecules by means of OH group (Fig. 7A), during the most part of trajectory, and with an average E^INT^ value of −12.5 kcal/mol (Fig. 8A). Moreover, we detected that, in the early stages of the simulation (13–14 ps), the OH group established a coordinate bond with Rh by forming the adduct [Cp*Rh(H_2_O)_3_(pC)]^2+^, in which the three water molecules and the OH function of **pC** coordinated the [Cp*Rh]^2+^ metal center (Fig. S6). In this structure, the X--[Cp*Rh]^2+^ PIE reaches the most negative values of the trajectory (∼−41.5 kcal/mol) while the X--wat1 interaction energy is positive due to the lacking of any H bond with the OH function, and to the occurrence of steric clashes (Fig. 8B).

Therefore, the analysis of the PIE values between water molecules and [Cp*Rh]^2+^ in the latter complex showed an important decrease of these interfragment energies, compared with values computed at 12 ps, suggesting a weakening of the Rh-water interaction. This data was corroborated by the increased wat-Rh bond lengths (Fig. S6), with highest value of 0.25 Å computed for wat3-Rh interaction. It is worth noting that wat3, found to be majorly affected by the O-coordination of the phenol moiety, was also detected as the first molecule replaced by OH in the MTD run1, thus we speculate that the **EC1** structures sampled at 13 and 14 ps of the MD run may recall the transition state structure for the release of the first water molecule. All these results indicate that the OH function of the phenol ligand is able to exert a strong influence on the stability of the first coordination sphere of the Rh(III) center, and it could attack the metal center in native conditions, i.e., even in absence of a pushing potential on the water oxygen.

On the contrary, for both **3MI** and **T** the weak interaction between the π-electrons of aromatic rings and H atoms of Rh-bound waters, characterizing the starting **EC2** and **EC3** structures, are rapidly break within the first ps of simulations and replaced by π-π interactions between Cp* ring and the aromatic scaffold of ligands (Fig. 7B and C) assuming a “reversed sandwich-like” configuration. The analysis of their E^INT^ and of the corresponding PIEs reflects these structural rearrangements of **EC2** and **EC3** (Fig. 8C–D and E–F, respectively). This binding mode, favored by hydrophobic and dispersion interactions, is stable over the most part of trajectories for both **3MI** and **T** with average E^INT^ of −12.5 and −6.2 kcal/mol, respectively (Fig. 8C and E). Notably, in this configuration, the ligand exchange reaction leading to PA cannot occur since the aromatic scaffold cannot attack the metal center.

## Discussion

The cationic [Cp*Rh(H_2_O)_3_]^2+^ complex can react in water, and in mild pH conditions, with an aromatic scaffold, X, leading to the exchange product, [Cp*Rh(η^6^-X)]^2+^. Designed to be employable to targeting the aromatic side chain of a.a. as Tyr, Trp, and Phe, this reaction has disclosed a high chemoselectivity for the conjugation of the Tyr phenol compared to the Trp and Phe side chains of GPCR peptides, as shown by the experimental and theoretical studies [17–19]. Indeed, many drugs acting on opioid receptors contain an aromatic ring; thus, their conjugation with [Cp*Rh]^2+^ might be explored as a possible strategy to modulate their pharmacological and pharmacokinetic profiles. Notably, the [(η^6^-Cp*Rh-Tyr^1^)-Leu-enkephalin]^2+^ showed an enhanced activity on the mu receptor compared with free peptide supporting the reliability of this strategy [53]. Although our previous studies [19] have provided evidences that the formation of [Cp*Rh(η^6^-Tyr)]^2+^ complexes is thermodynamically more favorable compared to either Trp and Phe, a deeper analysis of the structural aspects underlining such a chemoselectivity is lacking. In this work, to better characterize the main structural aspects involved in this specific ligand exchange process, we studied the reactivity of the aromatic scaffold **pC**, **3MI,** and **T**, mimetics of the aromatic side chain of Tyr, Trp, and Phe, respectively, by using DFT approaches, FMO analyses, and molecular dynamics simulations at tight-binding DFT level of theory.

The DFT investigation was focused on the conversion of the non-covalent adducts of [Cp*Rh(H_2_O)_3_]^2+^ with each of the a.a. mimetic scaffolds **pC**, **3MI,** and **T**, and provided the optimized structures of the encounter complexes **EC1**, **EC2**, and **EC3**, respectively, as well as those of the corresponding η^6^-complexes plus released water molecules, **PA1**, **PA2**, and **PA3**.

These calculations essentially reproduced the previously estimated reaction free energy trend [19] characterized by the most stable product adduct formed by **pC**, **PA1** with ∆G = −14.9 kcal/mol, while **PA2** and **PA3** were less stable, ∆G = −12.7 and ∆G = −7.6 kcal/mol , respectively.

The FMO3 analysis of DFT optimized structures of **PA1-3** complexes, by taking into account the presence of three, two, one or no water molecule in the second coordination sphere led us to estimate the effect of the water bulk on the interfragment interaction energy, thus, providing a direct assessment of the energy holding the stability of the η^6^-complexes. Indeed, we found that the interfragment interactions within the [Cp*Rh(η^6^-X)]^2+^ complexes are affected by the number of surrounding water molecules. In particular, the FMO outcomes indicated that when more than three explicit water molecules are present in the second coordination sphere, the **PA1** > **PA2** > **PA3** stability order is gained, consistently with the experimental findings [17, 18]. We envision that such an effect may be ascribed to the polarization of the OH group in the **pC** ligand, via the formation of H bonds with the bulk water molecules, that potentiates its +M effect, enhances the electronic density on benzene scaffold, and, in turn, strengthens the interaction with the [Cp*Rh]^2+^ scaffold. Although with a less intensity, this effect was also found for **3MI,** since OH as well as NH function of **3MI**, can act as H bond donor and the interaction with O atom of solvation water leads to rising the +M effect on aromatic scaffold.

This result suggests a possible role of solvation water also in the biological environment. Indeed, Tyr side chain can be found also in protein domains exposed to the solvent while Trp and Phe, due to their high hydrophobicity, are basically located in the buried regions. Thus, Tyr side chain should be more likely accessible from the bulk than Trp and Phe, and more exposed to the reaction with hydrophilic [Cp*Rh(H_2_O)_3_]^2+^ complex which is boosted by the action of solvation water molecules upon OH function increasing its +M effect and so to the **PA1** stability.

To detail the EC⟶PA process at room temperature, we performed meta-dynamics simulations at the tight-binding DFT level of theory, and applied the FMO3 analysis to representative snapshots of the calculated trajectories. These calculations revealed that the chemoselective η^6^-coordination of phenol compared to the indole and phenyl scaffolds is ascribed to the multifacet role of the OH group. Beside its +M effect on the electron density of the aromatic group, as evidenced by the DFT studies, the MTD simulations showed its ability to initiating and assisting the release of water molecules. Indeed, the OH function in **pC**, acting as H bond acceptor, allowed this aromatic ligand to interact with one or two water molecules coordinated at the [Cp*Rh]^2+^ scaffold. We envision that these interactions may strengthen the non-covalent interactions in **EC1**, but, concomitantly, also favor its rapid evolvement into the *η*^2^-complex. In detail, we hypothesized that the OH group, once non-covalently anchored at the [Cp*Rh]^2+^ scaffold, might directly attack the Rh center at the beginning of the ligand exchange reaction, by promoting a more effective replacement of the first water molecule than **3MI** or **T**. These latter aromatic molecules are devoid of either nucleophilic or H bond acceptor groups able to anchor at and/or attack the metallic center, and the conjugation reaction they give rise should rather start with the direct formation of the *η*^2^-complex and release of one water molecule.

The MTD simulation of the **EC1** species corroborated the formation the [Cp*Rh(H_2_O)_2_(κ^1^-pC)]^2+^ complex, its further evolvement into the transient *η*^2^ and *η*^4^ adducts before yielding the stable [Cp*Rh(*η*^6^-pC)]^2+^ complex. On the other hand, we found that *η*^4^ adducts are also transient complexes in the formation of **PA2** and **PA3**, even though these systems were mostly represented by the equilibrium of *η*^2^ and *η*^6^ forms. This evidence is in agreement with the DFT results indicating the lesser exergodicity in the formation of **PA2** and **PA3** compared to **PA1**.

The role of OH, and, more generally, of the non-covalent approach of the X ligand to the [Cp*Rh(H_2_O)_3_]^2+^ complex in the first stages of the ligand exchange reaction, was better described by the MD simulation of **EC1-3** complexes, without applying the MTD(RMSD) protocol. The Trp and Phe side chain mimetic scaffolds, **3MI** and **T**, were found to initially interact with the [Cp*Rh(H_2_O)_3_]^2+^ cation only by means of weak polar contacts (especially for **T**), while the phenol OH of **pC** established strong H bonds which last in the trajectory. As a consequence, the MD trajectory analysis showed the week polar π--HOH contacts are rapidly broken and **EC2** and **EC3** rearrange to a sort of reversed sandwich configuration between Cp* and aromatic rings of **3MI** or **T**, which is not favorable to the ligand exchange reaction since the aromatic scaffold cannot attack the Rh atom. As a result, the OH group not only enhanced the stabilization of the **PA1** structure compared with **PA2** and **PA3** by means of +M effect, augmented by the interaction with solvation water, but also contributed to dynamically sample a proper configuration of **EC1** where the reactive species, i.e., **pC** and [Cp*Rh(H_2_O)_3_]^2+^, are correctly oriented to initiate the **EC1**⟶**PA1** process. Thus, our results suggest that the OH group of phenol plays a crucial role in shaping the high chemoselectivity in the Cp*Rh(III)-conjugation of Tyr residues compared to Trp and Phe. Therefore, the combination of DFT studies, tight-binding DFT molecular dynamics simulations, and FMO analysis, here applied for the first time to study the ligand exchange reaction of metal complexes, provided an atomistic level of understanding of such chemoselectivity, and unveiling the importance of non-covalent interactions between **pC** and [Cp*Rh(H_2_O)_3_]^2+^ in both thermodynamics and kinetics of the formation of [Cp*Rh(η^6^-X)]^2+^ X=Tyr, Trp, and Phe bioconjugates.

Our further computational work will be devoted to improve the accuracy of this computational protocol by including explicit solvation water molecules and adopting the free energy decomposition analysis based on the FMO method combined with umbrella sampling molecular dynamics (US-MD) approach, recently proposed by Fedorov et al. [54].

These mechanistic aspects can be employed in the newly development of metal drugs based on the chemoselective [Cp*Rh(H_2_O)_3_]^2+^ targeting of Tyr residues in endogenous peptides or proteins.

**Fig. 9:**
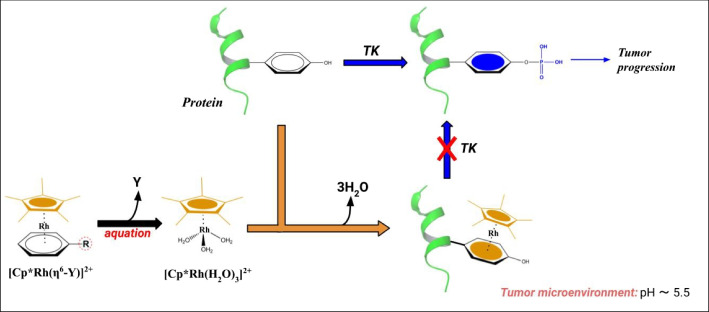
Schematic representation of possible antitumor action of [Cp*Rh(H_2_O)_3_]^2+^ inhibiting the phosphorylation of Tyr residue by means of a Tyr kinase enzyme

Indeed, the alterations of biological processes involving Tyr residues, such as Tyr phosphorylation [55] by means of Tyr-kinase enzymes, are found in several pathologies as in tumors [56]. Thus, the high selectivity conjugation of [Cp*Rh(H_2_O)_3_]^2+^ might be used to target the Tyr residues leading to [Cp*Rh(Tyr)]^2+^ and impending the action of Tyr-kinase enzymes (Fig. 9). Moreover, the target selectivity, a crucial feature of a drug, could be facilitated by the pH of tumor microenvironments, which is often around 5.5, thus, in the optimal range of pH values for the [Cp*Rh(Tyr)]^2+^ formation. It is worth noting that Rh(III)-containing complexes were already evaluated as inhibitors of pathological process kinase-dependent acting as direct protein-kinase (PK) inhibitors via hydrogen bonds with the ATP-binding site (e.g., phenylquinoline scaffold) [7]. In our view, the “prodrugging” of the [Cp*Rh]^2+^ scaffold into the cancer cells could represent a paradigm change since one can take advantage of Tyr conjugation selectivity to hit the protein substrates of PKs, and blocking the associated kinase-dependent pathways.

In analogy with CP, whose effective reactive species is obtained after aquation reaction, we propose that the [Cp*Rh(H_2_O)_3_]^2+^ complex could be in principle formed in the target biological compartment, i.e., the tumor tissue, by the hydrolysis of a precursor complex. Indeed, we envision that conjugated [Cp*Rh(η^6^-Y)]^2+^ complexes, bearing aromatic scaffolds (Y) that could be released to restore the active tris-aquo complex in the biological environment, may act as prodrugs. In this view, Y should be properly designed to enhance the rate and the thermodynamics of the aquation reaction, and possibly favor the tissue selectivity with less toxic effects. Indeed, Rh(III) complexes containing polypyridyl ligands (Y) demonstrated potent antitumor activity in vitro but their high toxicity impeded their application as drugs [57].

As shown in Fig. 9, after aquation reaction, the restored reactive species [Cp*Rh(H_2_O)_3_]^2+^, interacts with the Tyr side chain of the protein enhanced by acid pH of the tumor microenvironment, and the formed [Cp*Rh(Tyr)]^2+^ complex impedes the Tyr post-translational modification that potentially block the tumor progression.

Within the same paradigm, other Tyr post-translational modifications may also be hampered by the in cellula Cp*Rh(III)-conjugation. For instance, the Tyr sulfation, involved in the tuning of protein–protein interactions affecting the leukocyte adhesion, hemostasis and chemokine signaling processes [58], may potentially be affected by the Tyr-conjugation with [Cp*Rh]^2+^ and lead to innovative approaches, based on the disruption of PPIs, to treat related pathologies.

## Conclusion

The cationic complex [Cp*Rh(H_2_O)_3_]^2+^ has been demonstrated to react selectively with the phenolic side chain of Tyr, even in the presence of other aromatic a.a. side chains, such as Phe and Trp [17–19].

In this work, to deepen and clarify the mechanistic aspects of this high chemoselectivity, we studied in detail the conjugation of [Cp*Rh(H_2_O)_3_]^2+^ with three small molecules, **pC**, **3MI**, and **T**, mimetic of Tyr, Trp, and Phe side chains, respectively, by using the combined MTD/FMO3 and MD/FMO3 approaches.

Preliminary DFT calculations confirmed the highest stability of the [Cp*Rh(pC)]^2+^ complex compared with [Cp*Rh(3MI)]^2+^ and [Cp*Rh(T)]^2+^ and therefore the high selectivity for the Tyr side chain. The simulation of the formation of either the Cp*Rh(III)-conjugates or the non-covalent (aromatic scaffold )--[Cp*Rh(H_2_O)_3_]^2+^ encounter complexes were carried on via MTD or MD calculations at the tight-binding DFT level of theory.

The FMO analyses of the MTD and MD trajectories suggested that the higher selectivity for Tyr compared to Phe and Trp side chains might be related to the multifacet role played by the phenol OH group:

i) The +M effect of the OH function increases the electron density on the phenol ring, strengthening the η^6^-coodination in the final [Cp*Rh(pC)]^2+^ complex. Our calculations also showed that such an effect is significantly enhanced by the H bond with solvation water which polarizes the phenolic O-H bond increasing the partial negative charge on the O atom.
ii) At variance of **3MI** and **T** side chains, the phenolic OH group can act also as H bond acceptor thus establishing more effective H bond interactions with the aquo ligands of [Cp*Rh(H_2_O)_3_]^2+^ complex. These interactions probably favor the configurations of the non-covalent [Cp*Rh(H_2_O)_3_]^2+^ phenol EC amenable to the formation of the corresponding *η*^6^-complex, and therefore increasing the reaction rate.

Interestingly, the analysis of MTD trajectories also showed that not only the formation of η^6^-complexes proceeds via the interception of η^2^ and η^4^ intermediates, as previously found [19], but the **pC** compared to **T** and **3MI** demonstrated the most stable η^6^-coordination. On the other hand, the MTD trajectories also unveiled the η-complexes, mostly η^2^ and η^6^, and the non-covalent EC coexist and showed that the aquation of [Cp*Rh(η^6^-Y)]^2+^ complexes with *Y*=aromatic scaffold, is theoretically possible.

On the basis of abovementioned mechanistic aspects, we speculate that [Cp*Rh(η^6^-Y)]^2+^ complexes could be employed as prodrugs of the [CP*Rh]^2+^ to afford the chemoselective Cp*Rh(III)-conjugation of the Tyr side chains in cellula. In particular, we envision that newly designed [Cp*Rh(η^6^-Y)]^2+^ complexes can be employed to target the Tyr residues involved in biological/pathological processes, such as Tyr-kinase enzyme activity and protein–protein interactions.

Therefore, our results might contribute to extend the knowledge of the mechanism underlining the Tyr chemoselective conjugation with the [Cp*Rh(H_2_O)_3_]^2+^ complex and inspire the design of a new class of [Cp*Rh]^2+^-based metallodrugs.

### Supplementary information

ESM 1 The online version contains supplementary material available at https://doi.org/10.1007/s00894-023-05794-z.

## Data Availability

The 3D coordinates of the optimized structures, obtained by sampling each 1 ps of the **EC1**, **EC2**, and **EC3** trajectories (MTD and MD calculations) and used as input geometries for FMO3 calculations, are available in the xyz file format at the following link 10.5281/zenodo.8417065.
