# Supplementary material for: A computational insight on the aromatic amino acids conjugation with [Cp*Rh(H2O)3]2+ by using the meta-dynamics/FMO3 approach
Source: J Mol Model. 2023 Dec 12;30(1):4. doi: 10.1007/s00894-023-05794-z (PMC10713709; doi:10.1007/s00894-023-05794-z)
Supplement: Supplementary file 1 — The online version contains supplementary material available at https://doi.org/10.1007/s00894-023-05794-z. [file 894_2023_5794_MOESM1_ESM.pdf]

Supporting Information

A computational insight on the aromatic amino acids conjugation with $[Cp^*Rh(H_2O)_3]^{2+}$ by using the Meta-dynamics/FMO3 approach.

Roberto Paciotti[1]*, Alessandro Marrone[1]

[1] Department of Pharmacy, Università "G. D'Annunzio" Di Chieti-Pescara, Chieti, I-66100, Italy.

*corresponding to: r.paciotti@unich.it

Table of Contents

Fig. S1 2
Table S1 2
Table S2 3
Fig. S2 3
Fig. S3 4
Fig. S4 5
Table S3 5
Fig. S5 6
Fig. S6 7

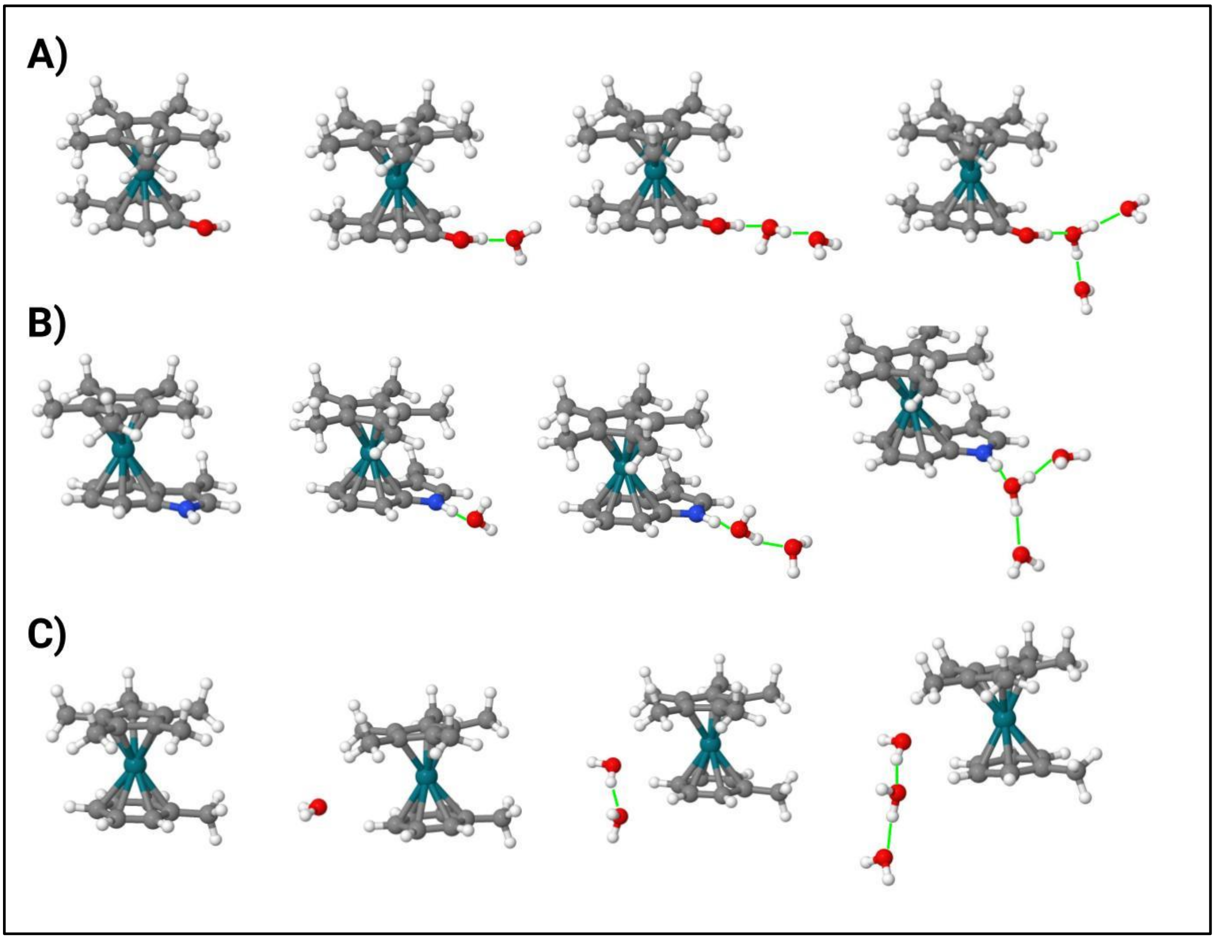

Fig. S1 Lowest energy structures computed at B3LYP//ωB97X-D/6-311+G(d,p) of A) $[Cp^*Rh(pC)]^{2+}$, B) $[Cp^*Rh(3MI)]^{2+}$ and C) $[Cp^*Rh(T)]^{2+}$ along with the corresponding PAs with one, two and three explicit solvation waters. Hydrogen bonds are presented by green lines

Table S1 X--$[Cp^*Rh]^{2+}$ PIE values by varying the number of explicit waters (from zero to three) including X, $[Cp^*Rh]^{2+}$, and water molecules in separated fragments and including X and water directly interacting via H bond in the same fragment. All energy values are in kcal/mol

**number of explicit water molecules**	**X--$[Cp^*Rh]^{2+}$ PIE**				
	5 fragments			*X and H_2O in the same fragment*	
	pC	***3MI***	***T***	***pC***	***3MI***
0	-114.6	-121.8	-121.7	-114.6	-121.8
1	-118.6	-124.0	-124.5	-120.9	-124.6
2	-121.0	-125.7	-125.1	-123.5	-127.0
3	-125.6	-128.3	-126.6	-130.6	-129.3

Table S2 Average X--[Cp*Rh]$^{2+}$ PIE values with the corresponding EDAs computed considering stable η^6-PAs obtained in run 1 and 2. Energy and CT values are reported in kcal/mol and elementary charge units, respectively

**Complex**		**PIE**	**E^{es}**	**E^{ex}**	**E^{ct}**	**E^{disp}**	**E^{sol}**	**CT**
PA1	*average*	-122.0	-196.4	245.1	-130.9	-55.0	15.2	-0.445
	std	1.6	4.3	7.0	5.0	0.7	2.2	0.012
PA2	*average*	-130.4	-209.4	241.4	-127.7	-56.5	21.9	-0.400
	std	11.8	16.5	29.5	18.7	5.9	1.2	0.079
PA3	*average*	-128.8	-198.2	240.6	-128.3	-56.2	13.4	-0.420
	std	1.1	3.1	6.3	3.9	0.6	1.0	0.007

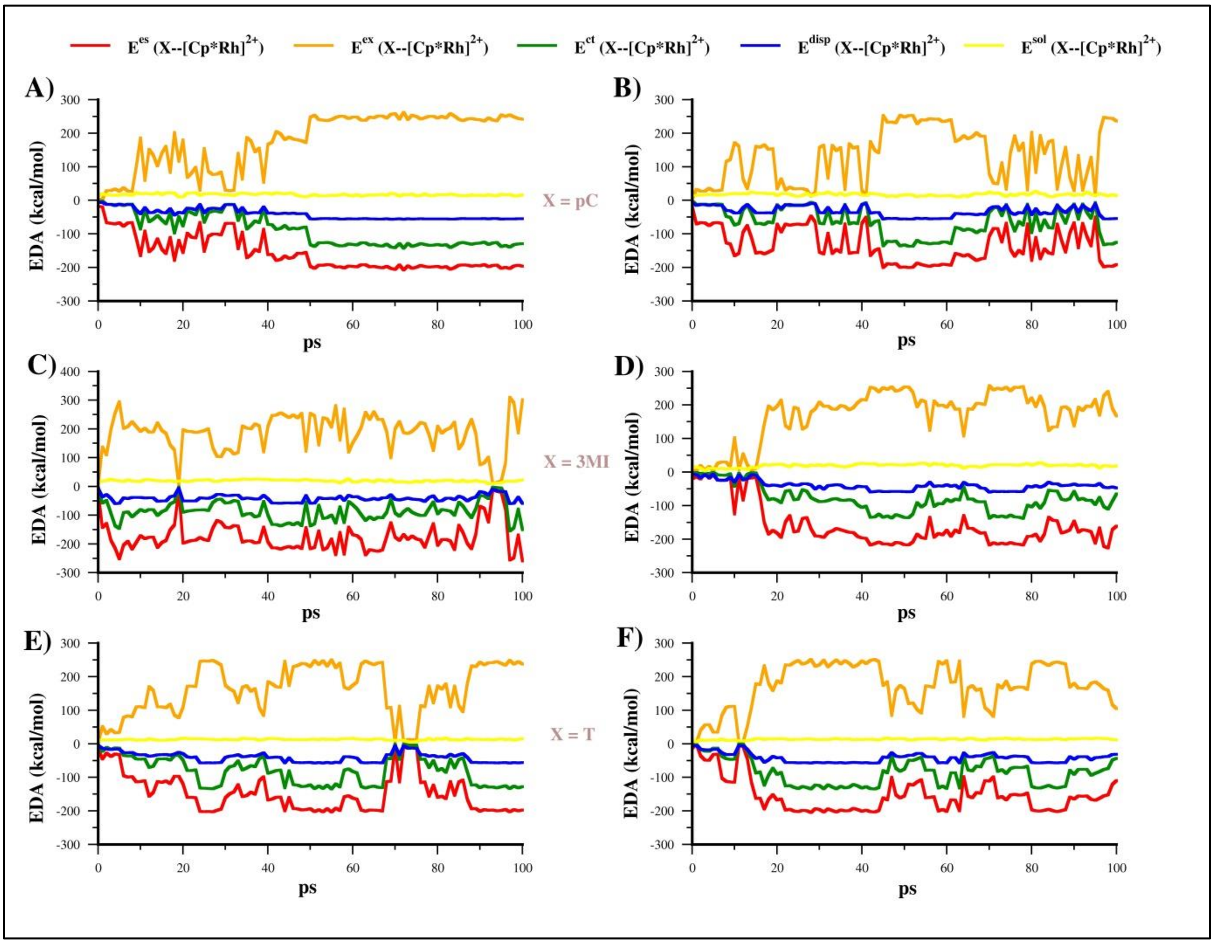

Fig. S2 EDA of X--[Cp*Rh]$^{2+}$ PIEs computed for sampled structures of MTD(RMSD) run 1 and 2 for **EC1** (a and b), **EC2** (c and d) and **EC3** (e and f)

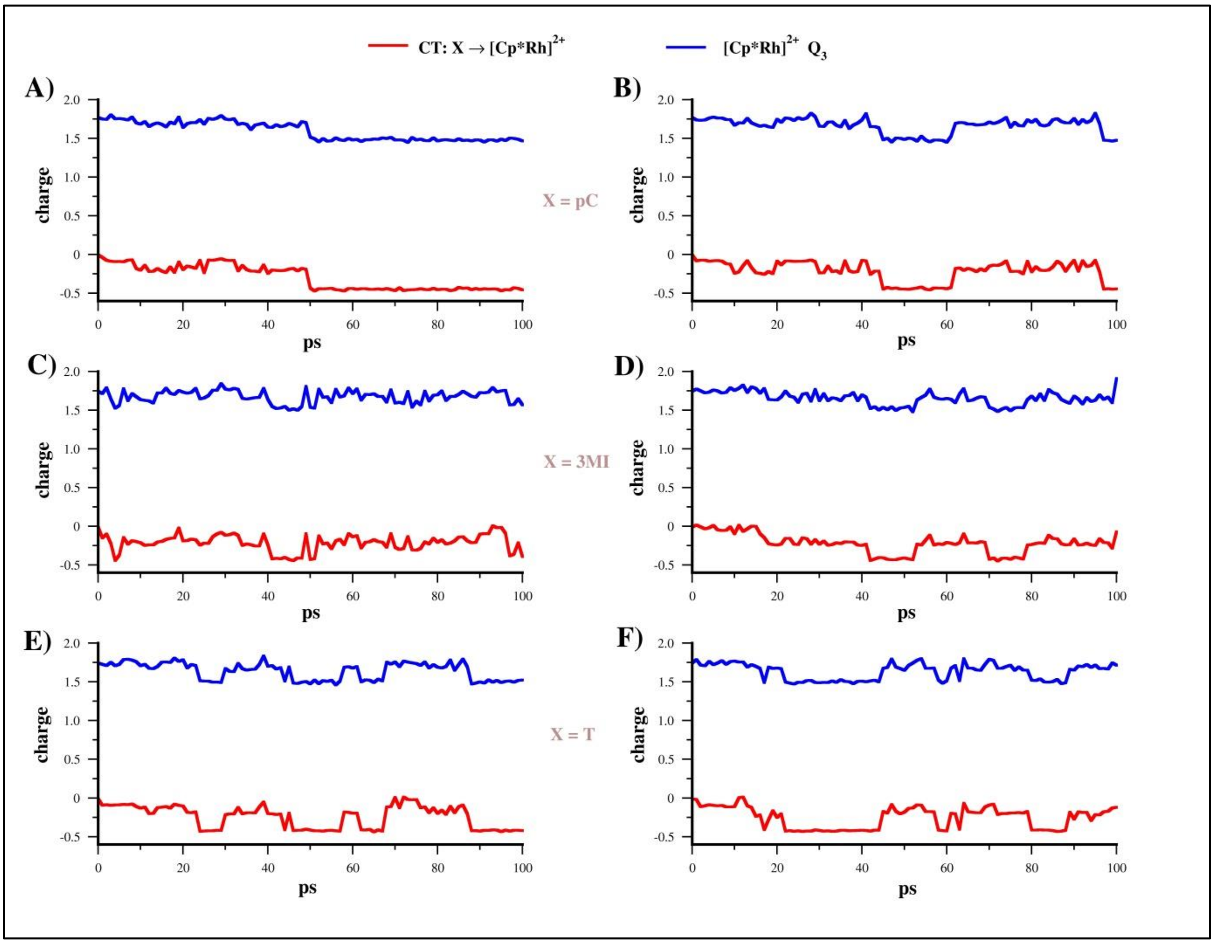

Fig. S3 CT from X to $[Cp^{*}Rh]^{2+}$ (red line) and Q_3 of $[Cp^{*}Rh]^{2+}$ (blue line) computed for sampled structures of MTD(RMSD) run 1 and 2 for **EC1** (a and b), **EC2** (c and d) and **EC3** (e and f)

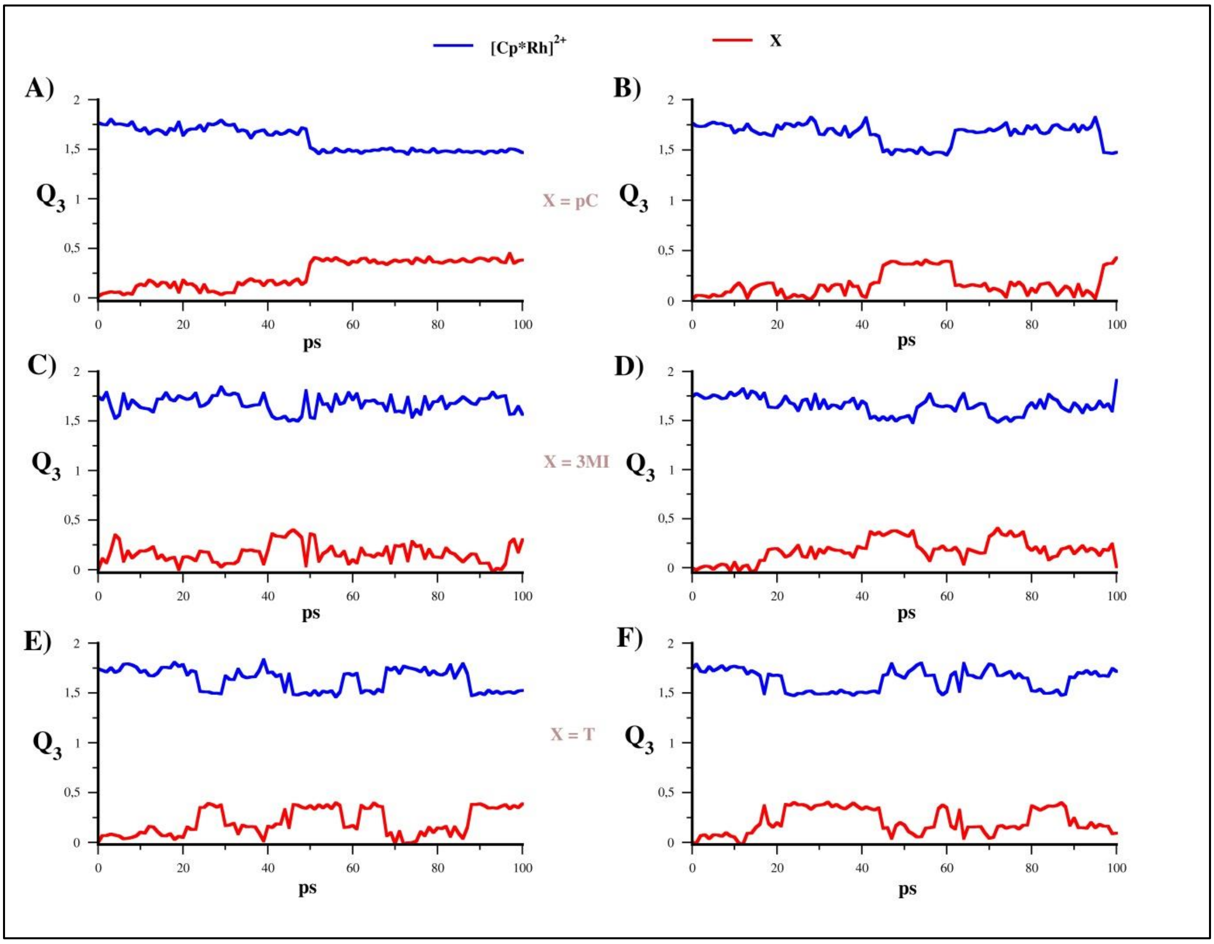

Fig. S4 Q_3 values of X and $[Cp^*Rh]^{2+}$ (red and blue line, respectively) computed for sampled structures of MTD(RMSD) run 1 and 2 for **EC1** (a and b), **EC2** (c and d) and **EC3** (e and f)

Table S3 E^{INT} values computed for **pC**, **3MI** and **T** in **EC1**, **EC2** and **EC3** structures optimized at B3LYP/6-311+G(d,p)//ωB97X-D/6-311+G(d,p) level of theory. All energy values are in kcal/mol

**Structure**	$\mathbf{E^{INT}}$
pC	-17.5
3MI	-14.4
T	-10.3

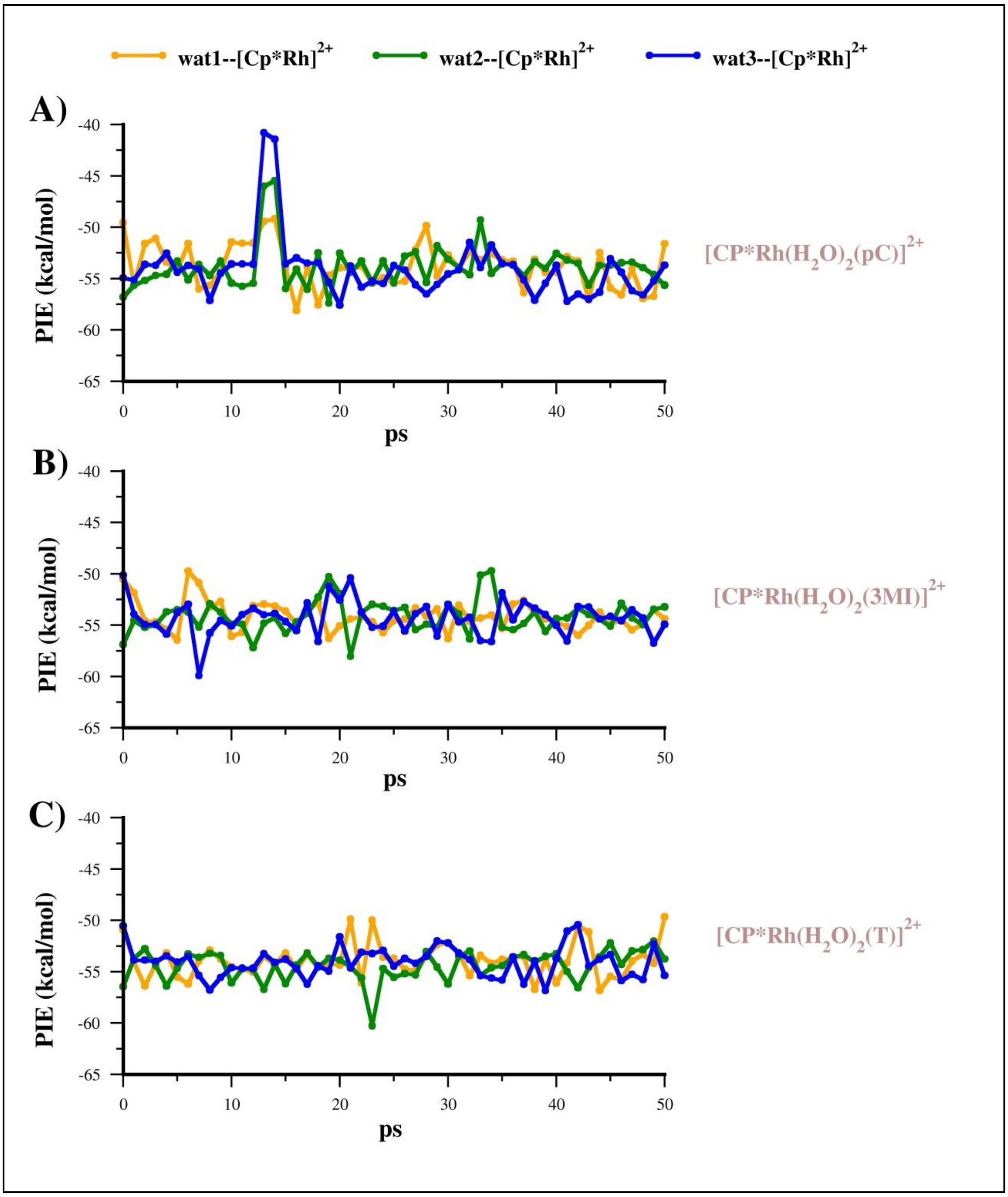

Fig. S5 PIEs values of wat1--$[CP^*Rh]^{2+}$ (yellow line), wat2--$[CP^*Rh]^{2+}$ (green line) and wat3--$[CP^*Rh]^{2+}$ (blue line) interactions computed for the sampled structures of MD simulations of A) **EC1**, B) **EC2** and C) **EC3**

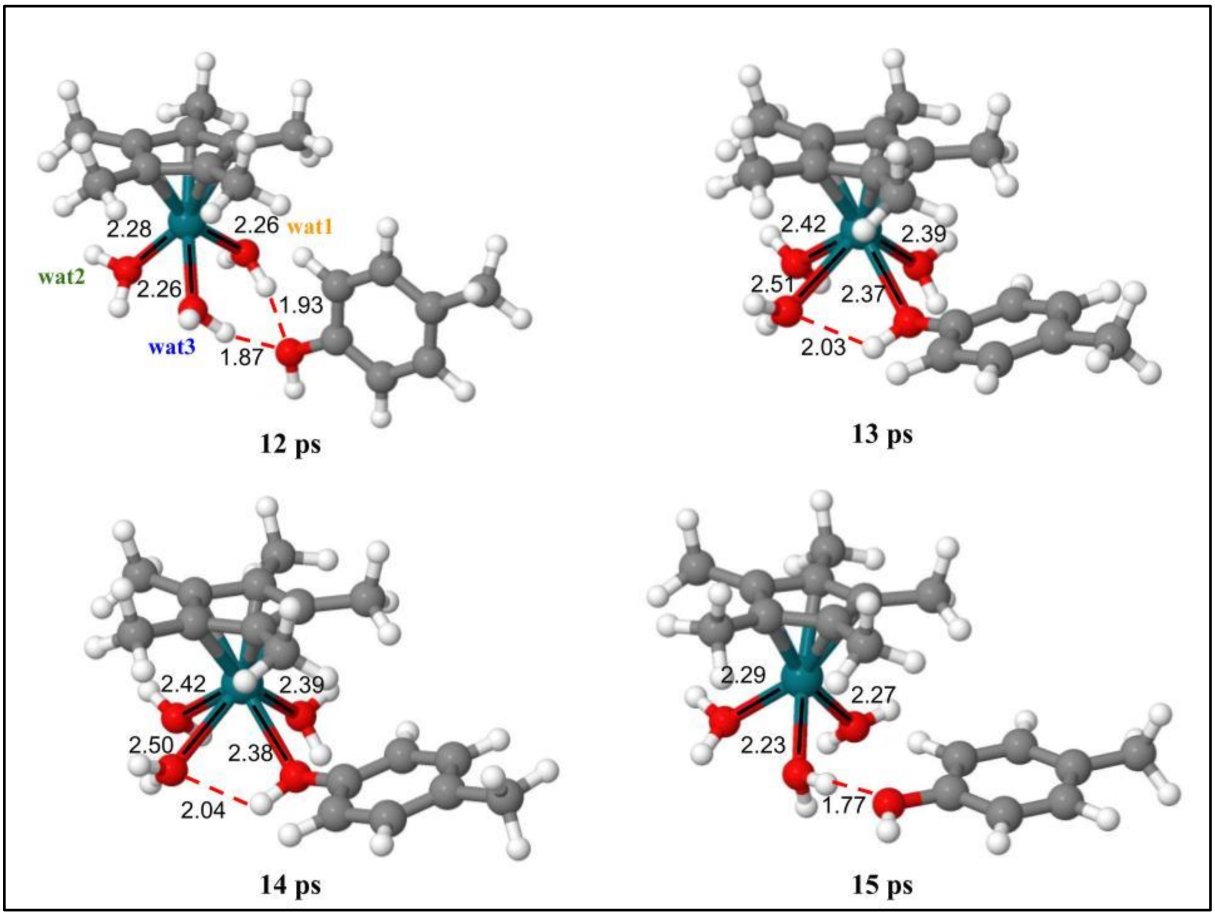

Fig. S6 EC1 structures of MD simulations sampled at 12, 13, 14 and 15 ps. Hydrogen bonds and Rh-O coordinate bonds are reported in red dashed lines and in black lines, respectively
